# Role of microglia in diabetic neuropathic pain

**DOI:** 10.3389/fcell.2024.1421191

**Published:** 2024-07-29

**Authors:** Qian Wang, Yilin Xie, Shichao Ma, Hongliang Luo, Yue Qiu

**Affiliations:** ^1^ Department of Endocrinology and Metabolism, Jiujiang Hospital of Traditional Chinese Medicine, Jiujiang, Jiangxi, China; ^2^ School of Ophthalmology and Optometry, Jiangxi Medical College, Nanchang University, Nanchang, Jiangxi, China; ^3^ Department of Gastrointestinal Surgery, The Second Affiliated Hospital, Jiangxi Medical College, Nanchang University, Nanchang, Jiangxi, China

**Keywords:** diabetes, neuropathic pain, microglia, mechanism, treatment

## Abstract

Approximately one-third of the patients with diabetes worldwide suffer from neuropathic pain, mainly categorized by spontaneous and stimulus-induced pain. Microglia are a class of immune effector cells residing in the central nervous system and play a pivotal role in diabetic neuropathic pain (DNP). Microglia specifically respond to hyperglycemia along with inflammatory cytokines and adenosine triphosphate produced during hyperglycemic damage to nerve fibers. Because of the presence of multiple receptors on the microglial surface, microglia are dynamically and highly responsive to their immediate environment. Following peripheral sensitization caused by hyperglycemia, microglia are affected by the cascade of inflammatory factors and other substances and respond accordingly, resulting in a change in their functional state for DNP pathogenesis. Inhibition of receptors such as P2X reporters, reducing cytokine expression levels in the microglial reactivity mechanisms, and inhibiting their intracellular signaling pathways can effectively alleviate DNP. A variety of drugs attenuate DNP by inhibiting the aforementioned processes induced by microglial reactivity. In this review, we summarize the pathological mechanisms by which microglia promote and maintain DNP, the drugs and therapeutic techniques available, and the latest advances in this field.

## Introduction

In recent years, the number of cases and prevalence of diabetes has been steadily increasing. According to the latest data by WHO, approximately 422 million people worldwide have diabetes (World Health Organization, 2024). The global prevalence of diabetes is projected to reach 780 million by 2045 (Sun et al., 2022). Diabetes has become a typical disease that poses a significant threat to human health. Each year, global mortality because of diabetic complications equals approximately 1.5 million (World Health Organization, 2024). Complications of diabetes include diabetic nephropathy, retinopathy, vascular disease, and peripheral neuropathy, and more than half of the patients with diabetes worldwide experience diabetic neuropathy (Saeedi et al., 2019). Diabetic neuropathy is associated with debilitating physical effects to patients and a significant economic burden. The costs associated with patients suffering from diabetic peripheral neuropathy exceed those associated with non-diabetic peripheral neuropathy patients in terms of the number of medications used and total annual medication costs (Pan et al., 2023). Diabetic neuropathy can be painful or non-painful (Gylfadottir et al., 2019). In this review, we focus on painful diabetic neuropathy (hereafter referred to as diabetic neuropathic pain, DNP), characterized by sharp, burning, electric shock-like, and evoked pain (Galer et al., 2000). Among diabetic neuropathy types, DNP is the most severe in terms of impact on the daily lives of affected patients (Gylfadottir et al., 2022; Tesfaye et al., 2023). Therefore, clarifying the pathological mechanism of DNP and its treatment strategies is essential.

There is much research into the pathological mechanisms of DNP. In a streptozotocin (STZ)-induced diabetes model, a study demonstrated that the reactive microglial marker ionized calcium binding adapter molecule 1 (IBA-1) is highly expressed in the spinal cord of diabetic mice, implying an inextricable link between DNP and microglia (Hwang et al., 2024). The involvement of microglia in the pathological mechanisms of DNP and its therapeutic methods have become hot topics of research in recent years, suggesting a close correlation between spinal microglia and the pathogenesis and progression of DNP.

However, the mechanism underlying the involvement of microglia in DNP is unclear. We have therefore explored in detail the hypothesized pathological mechanisms and therapeutic approaches regarding the involvement of microglia in DNP.

## Mechanism of diabetic neuropathy

After diabetes development, typical symptoms including excessive eating, drinking, urinating, and weight loss ensue. Hyperglycemia is the most severe sign of diabetes, leading to the production of toxic metabolites, which at the cellular level are characterized by the accumulation of mitochondrial dysfunction, metabolic switch, oxidative stress, and axonal degeneration (Fernyhough, 2015). During hyperglycemia, abnormalities in polyols, hexosamine, and protein kinase C (PKC) pathways lead to inflammation and increased levels of reactive oxygen species (ROS) (Feldman et al., 2017). These effects can lead to nerve damage, which in turn can result in neuropathy. In diabetic neuropathy, the initial occurrence involves functional changes in C fibers, such as initial degeneration and subsequent regeneration, which lead to pain and hyperesthesia, and may cause microglial reactivity (Green et al., 2010; Ota et al., 2021). Diabetic polyneuropathy is the most common type of diabetic neuropathy, mainly manifested as mixed fiber polyneuropathy, whereas some are pure small fiber or large fiber polyneuropathies, with simultaneous involvement of Aβ-, Aδ-, and C fibers (Galosi et al., 2021). Neuropathic pain in majority of the patients is clearly associated with small fiber damage (Galosi et al., 2021). Patients with DNP may also experience phenotypic alterations in neuronal gene expression or complete loss of neurons, leading to increased inflammation within the dorsal root ganglion (DRG) and decreased levels of neuronal transcriptional material (Hall et al., 2022). DRG neurons exposed to systemic metabolic and hypoxic stressors are more susceptible to damage (Feldman et al., 2017), and DRG neuron dysfunction leads to worsening of DNP (Xie et al., 2022). In diabetes, hyperglycemia causes nerve damage and discharges messages to the dorsal horn neurons of the spinal cord to induce central sensitization of the spinal cord (Zhu et al., 2019; Eid et al., 2023). When diabetic neuropathy occurs, microglia respond and release signaling molecules that cause neuro-inflammation and are therefore involved in DNP.

## Role of microglia in neuropathy and DNP

In recent years, many researchers have emphasized the diverse and critical roles of microglia in brain development, homeostasis, and pathology, especially for the central nervous system (CNS).

Microglia account for 5%–20% of the total neuroglia in the mouse brain (Lawson et al., 1990), 0.5%–16.6% of the total amount of all human CNS cells, and are more plentiful in white matter tracts than in gray matter (Mittelbronn et al., 2001). Microglia are produced by myeloid progenitor cells in the yolk sac of a developing mouse before embryonic day 8 (Ginhoux et al., 2010). Microglia are not only the central integrators of neurological disease risk, but also critical mediators in developing neurological pathology (Wright-Jin and Gutmann, 2019). Microglial development requires colony-stimulating factor 1 (CSF-1) signaling, the CSF-1 receptor articulating protein DAP12, and the interferon-regulatory factor-8 (Wolf et al., 2017). At individual microglial cell level, the landscape can change dramatically in weeks. Microglial population turnover is a highly dynamic process, with a very high proliferation rate in the mouse and human brain. With age, the number of microglia and contact sites with dopamine neurons increase, but their complexity decreases (Shaerzadeh et al., 2020).

Microglia are continuously dynamic rather than strictly quiescent in their physiological state. They are recognized as highly dynamic and plastic cells that exhibit multivariate morphological/ultra-structural, transcriptional, metabolic, and functional states in the CNS under both healthy and pathological conditions (Paolicelli et al., 2022). Microglia have strong plasticity in the CNS and can respond quickly to subtle transformations in the surrounding environment. Microglial responses to pathological conditions, such as trauma, involve them becoming hypertrophic, and the thin processes are pulled back into their soma, resulting in a round, amoeboid-like appearance (Perry, 2010).

Microglia regulate existing myelin through TGFβ1, which maintains the structural integrity of myelin while preventing demyelination and myelin hyperplasia, and a lack of microglia is detrimental to myelin health (McNamara et al., 2023). Myelin wraps around neuronal axons to maintain proper electrical impulse propagation (McNamara et al., 2023), which is essential for maintaining CNS function. Additionally, reactive microglia are involved in phagocytosis, whereas prolonged microglial activation causes neuronal damage (Yuan et al., 2019). Microglia also release brain-derived neurotrophic factor (BDNF) to promote synaptic remodeling and the onset of pain hypersensitivity after peripheral nerve injury (Huang et al., 2021). The study found that neonatal administration of STZ (nSTZ) increased OX-42 immunoreactivity and the percentage of hypertrophied and ameboid microglia in the spinal dorsal horn (Barragán-Iglesias et al., 2018). Researchers found that GYY4137 produced neuro-protective effects in diabetic rats, and abnormal pain as well as mechanical hyperalgesia were improved in the treated group. At the cellular level, treatment with GYY3137 reduced the number of microglia in the white and gray matter of the spinal cord, and the researchers believe that GYY4137 may alleviate DNP by reducing the number of reactive microglia in the spinal cord (Shayea et al., 2020). This suggests that microglia may be involved in the pathogenesis of DNP.

## Microglial involvement in DNP pathogenesis

DNP is caused by peripheral nerve damage due to successive metabolic disruptions of hyperglycemia, which manifests as peripheral neuropathic pain. The neuropathic pain mechanisms involve ion channel alteration, peripheral sensitization and central sensitization.

Hyperglycemia in the body has a direct toxic effect on the peripheral nerves and causes peripheral microvascular lesions, resulting in impaired nutrient supply to nerve cells. Peripheral sensitization caused by a cascade of inflammatory responses generated by the peripheral immune cells after nerve injury and central sensitization caused by reactivity of central glial cells are essential factors in the development and progression of neuropathic pain.

Peripheral sensitization represents the increased reactivity and decreased threshold of peripheral neurons to stimulate their receptive field (International Association for the Study of Pain, 2017). In patients with diabetes, primary afferent neurons, such as unmyelinated C-fibers and small-myelinated Aδ-fibers, are continuously stimulated by hyperglycemia. Multiple pathways release inflammatory factors such as TNF-α, IL-1β, and IL-6, which cause reactivity of transient receptor potential vanilloid type 1 and the release of substance P (SP) and calcitonin gene-related peptide (CGRP). The SP and CGRP release activates peripheral immune cells and promotes inflammatory factor release (Zang et al., 2023). Macrophages are typical representatives of peripheral immune cells, which act as house-keeping cells and play a role in immune detection through phagocytosis and cytokine secretion. In response to changes in environmental signals, macrophages shift between M1 pro-inflammatory and M2 anti-inflammatory phenotypes and function accordingly (Kim et al., 2019). When primary neurons are damaged, macrophages release nociceptive mediators and inflammatory cytokines in conjunction with satellite glial cells to enhance and maintain neuropathic pain (Yang et al., 2023a).

Central sensitization is the increased reactivity of nociceptive neurons in the central nervous system to their normal or subliminal afferent inputs (International Association for the Study of Pain, 2017). Reactive microglia in the dorsal horn of the spinal cord play an important role in initiating central sensitization. In this review, we have focused on the mechanisms by which microglia exert their effects in DNP.

The involvement of microglia in DNP can be divided into two stages: how diabetes activates microglia and how reactive microglia participate in and maintain DNP.

### Association of DNP with microglia reactivity

#### Hyperglycemia

Hyperglycemia enhances triglyceride levels and PKC reactivity in endothelial cells and large arteries, leading to an increase in arachidonic acid release and prostaglandin E2 production, which inhibits Na^+^, K^+^, and adenosine triphosphatase, potentially slowing nerve conduction. Hyperglycemia also triggers vascular endothelial cell dysfunction, leading to microvascular lesions causing nerve trophic disorders, further causing peripheral nerve damage and necrosis, altering the homeostatic microenvironment of the CNS and thus affecting the microglia. Hyperglycemia directly increases the number of microglia involved in DNP development (Lanlua et al., 2020). When hyperglycemia causes localized inflammation in the body, adenosine triphosphate (ATP) accumulates at the inflammation site (Di Virgilio et al., 2020). Hyperglycemia also causes mitochondria to produce large amounts of superoxide and increase the flux of polyol hexosamine and PKC channels, leading to an increase in microglial ROS production (Sharma et al., 2012). Excessive ROS in microglial cells enhance the role of NLR family pyrin domain containing 3 (NLRP3) (Zhou et al., 2011). When microglia are in a hyperglycemia-induced inflammatory environment, ATP levels in the surrounding environment are elevated (Koepsell, 2020). Inflammatory factors produced by peripheral neurons can bind to Toll-like receptors (TLRs) on microglia; hyperglycemia also prolongs NF-κB activation induced by lipopolysaccharide (LPS) binding to TLRs (Hung et al., 2022); and ATP released by stressed or injured cells or inflammation can bind to P2XR on microglia. By activating the p38 MAPK and nuclear factor κB (NF-κB) pathways, microglia can synthesize and release pro-inflammatory cytokines, such as IL-1β, IL-6, IL-18, and TNF-α, which are involved in pain signaling (Liu et al., 2021).

#### P2XR

P2XR is a ligand-gated cation channel activated by extracellular ATP signaling, and seven separate genes encoding P2X subunits have been identified and named P2X1-P2X7, who belong to a larger family, the purinergic receptors (Oken et al., 2022).

P2X4R is widely expressed in most cell types of the central and peripheral nervous systems, including neuronal and immune cells, especially microglia (Montilla et al., 2020). On microglia, the activation of the microglial intracellular inflammatory group NLRP3 through the binding of ATP to P2X4R leads to the maturation and release of inflammatory factors. It has been demonstrated that modulation of the P2X4R and NLRP3/ILL-1 inflammatory pathways in microglia by electroacupuncture resulted in significant pain relief (Zhou et al., 2024). Such a response is also reflected in P2X7 receptor (P2X7R), and inhibition of P2X7R expression by electroacupuncture can also inhibit the secretion of inflammatory factors by microglia and alleviate the inflammatory response (Lin et al., 2024).

#### TLR4

TLRs are an important class of protein molecules involved in non-specific immunity. Toll-like receptor 4 (TLR4), expressed on the surface of microglia, plays an important role in mediating LPS-induced microglial reactivity and inflammatory responses (Zusso et al., 2019).

TLR4 induces the recruitment of myeloid differentiation factor 88 and the transcriptional process of NF-κB in neurons and glial cells, which produces a variety of cytokines such as TNF-α and IL-1β and contributes to pain induction and persistence (Kawai and Akira, 2010; Sun et al., 2023). Hyperglycemia prolonged the activation of the microglial NF-κB pathway induced by LPS binding to TLR4 and secreted more inflammatory factors, which exacerbated the inflammatory response (Hung et al., 2022).

#### p38MAPK

p38 is an intracellular signaling lineage-activated protein kinase (MAPK) that is divided into four isoforms, p38α–δ. The MAPK pathway is involved in neuron-microglia signaling and plays an important role in pain generation (Ji et al., 2009). During DNP, pro-inflammatory substances can activate the p38MAPK signaling pathway, producing pain signals (Ni et al., 2016). p38MAPK inhibition can block pro-inflammatory cytokine secretion (Brown et al., 2015). Activation of p38MAPK in microglia has been demonstrated in animal pain models but not in non-painful diabetic animals (Suzuki et al., 2011). Diabetic pathogenic nerve pain can be effectively relieved by inhibiting p38 phosphorylation of in microglia (Zhang T. T. et al., 2018).

#### NLRP3

NLRP3 is a filamentous signaling platform consisting of three components (sensor, adapter, and effector) that mediate the inflammatory response by promoting the maturation of pro-inflammatory factors. Activation of the NLRP3 inflammatory vesicle pathway requires two steps; first, NLRP3 expression can be initiated by cytokines involved in the inflammatory response and is transcriptionally upregulated upon activation of NF-κB or other transcription factors (Bauernfeind et al., 2009). NLRP3 can then be activated by ATP (Perregaux and Gabel, 1994). Activated NLRP3 becomes an inflammatory complex through aggregation, and caspase-1 in the inflammatory complex cleaves cytokine precursors to produce mature forms such as IL-1β and IL-18 (Sharma and de Alba, 2021).

High expression of microglial NLRP3, ASC, and caspase-1 proteins and significant elevation of serum cytokines IL-1β, IL6, IL18, and TNF-α were found in the STZ-induced diabetic mouse model (Li et al., 2021b). It has also been demonstrated that by inhibiting the activation of NLRP3 in microglia, the production of IL-1β can be reduced and neuroinflammation under diabetes can be suppressed (Iwasa et al., 2023).

### Mediation of factors release by reactive microglia and its role in DNP

TNF-α, IL-6, and IL-1β released by microglia act on chemokine receptors in neurons, causing phospholipase C increase and PKC activation, which in turn, causes phosphorylation and activation of TRPV. As a result, the sensory neurons become hyper-responsive, and ROS and nitrogen radicals causing cytotoxic damage to the nerves are produced (Bhandari et al., 2021). We thus found that reactive microglia damage nerves by releasing these inflammatory factors during DNP, suggesting that microglia contribute to and maintain the development of DNP.

#### Interleukin factors

IL-1β is an interleukin-1 subtype that plays a pro-inflammatory role in the body. The NLRP3 inflammasome cleaves pro-IL-1β to active IL-1β (Lamkanfi and Dixit, 2014), which can bind to receptors on cells such as neurons and cooperate with other cytokines to trigger a spectrum series of signaling events that result in the exacerbation of inflammatory cascade responses within the central nervous system (CNS) (Song et al., 2017). Using high glucose (35 mM) treated BV2 cells (immortalized primary microglia) to simulate mimetic hyperglycemia *in vitro*, IL-1β transcription and expression levels were elevated after 8 h (Li et al., 2021a). This suggests that reactive microglia in a hyperglycemic environment participate in DNP development by releasing IL-1β, causing nerve damage.

IL-6 is another cytokine involved in the inflammatory response and in the process of diabetic peripheral neuropathy (Rahman et al., 2016). In a streptozotocin (STZ)-induced diabetes model, the study revealed that Mdivi-1 inhibited microglia from releasing inflammatory factors like IL-6, which can attenuate inflammation and neuron cell apoptosis (Chung et al., 2022).

IL-18 is a potent inflammatory cytokine expressed in microglia in the dorsal horn of the spinal cord. IL-18 receptor (IL-18R) is expressed mainly in astrocytes and the IL-18/IL-18R axis mediates the interaction between microglia and astrocytes. Unlike IL-1β, IL-6, and TNF-α, IL-18 is only released by microglia in the spinal cord and activates the NF-κB signaling pathway in astrocytes *via* IL-18R, triggering a cascade of responses involved in chronic pain development (Miyoshi et al., 2008; Ju et al., 2024). Serum IL-18 levels were significantly elevated and IL-18R was significantly upregulated in patients with type II diabetes mellitus compared to non-diabetic controls (Abhilasha et al., 2023). This shows the involvement of microglia in DNP by releasing IL-18 acting on IL-18R on astrocytes.

#### TNF-α

TNF-α is mainly produced by microglia (Welser-Alves and Milner, 2013), and is recognized as a key player in the reactivity and morphological changes of microglia in the spinal cord caused by peripheral nerve injury (Andrade et al., 2011). TNF-α may be an independent risk factor for peripheral neuropathy in patients with impaired glucose regulation (Li et al., 2017). TNF-α protein levels were significantly increased in a diabetic group of mice compared to those in the control group (Ghazipour et al., 2022). This suggests that during the DNP development, reactive microglia exacerbate peripheral nerve damage by releasing TNF-α to promote DNP.

#### BDNF

BDNF is a member of the trophic factor nerve growth factor family, which acts on motor and sensory neurons in the peripheral nervous system. A concomitant increase in the expression of BDNF was observed alongside an increased number of reactive microglia in the spinal cord of STZ-induced diabetic model (Ismail et al., 2020), suggesting that BDNF and microglia are involved in the process of DNP. BDNF promotes microglial reactivity and IL-1β and TNF-α release, which exacerbates neuro-inflammation and leads to mechanical allodynia through BDNF-TrkB-p38/JNK signaling (Ding et al., 2020).

#### IGF-1

Insulin-like growth factor-I (IGF-1) is a multifunctional protein playing a significant role in the development and maturation of CNS, including promoting neuronal survival and synaptic growth (Dyer et al., 2016; Falomir-Lockhart et al., 2019). IGF is mainly derived from microglia in the brain (Suh et al., 2013). Microglial IGF-1 levels were reduced in type 1 diabetic mice (T1DM) mice, and the activation of spinal IGF-1 signaling maintained microglial IGF-1 expression in the spinal cord of diabetic mice and reduced neuro-inflammation (Chen et al., 2022).

#### Chemokine (C-X-C motif) ligand (CXCL)12

**FIGURE 1:**
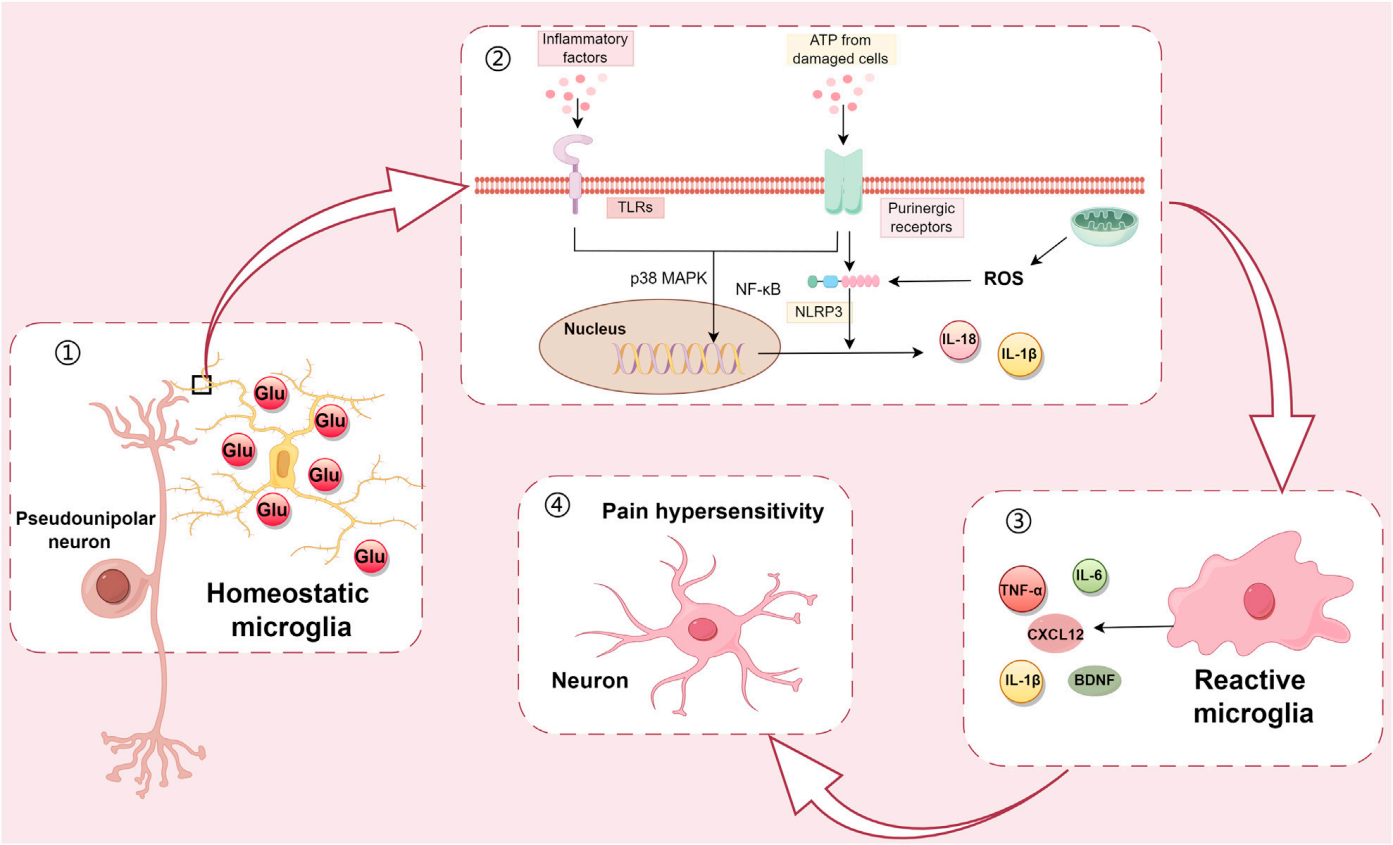
Microglial response to the hyperglycemic environment at the cellular level. Microglia located around neurons in the dorsal horn of the spinal cord are not in an absolutely resting state. Instead, microglia are constantly sensing their surroundings and responding accordingly to regulate their functional state. ① Under hyperglycemia-induced peripheral sensitization, microglia are exposed to ATP, inflammatory factors and other substances produced by cascade reactions; ② Through the signaling including purinergic receptors and toll-like receptors, NLRP3 pathway activation is induced, and through the p38 MAPK/NF-κB signaling pathway, the microglia respond accordingly; ③ Changes to the morphology of microglia such as cellular hypertrophy and increased thickness occur, resulting in an amoeba-like morphology. At the same time, microglia produce pro-inflammatory cytokines such as IL-1β, IL-6, TNF-α, and BDNF; ④ Finally, these cytokines act on neurons to cause pain hypersensitivity. All of the above processes illustrate that microglia are involved in the development and progression of DNP.

CXCL12-CXCR4 chemokine signaling plays a key role in regulating various neurodevelopmental processes and modulating synaptic plasticity (Li and Ransohoff, 2008). In the study of a mouse diabetic model established by injection of STZ, it revealed that immunostaining showed that CXCL12 was invariably co-labeled with IBA-1, and reactive microglia can release CXCL12, which subsequently acts on neuronal CXCR4 to cause neuronal hyperactivation (Song et al., 2023). This study demonstrates the involvement of microglial reactivity in the development of diabetic neuropathy by up-regulating CXCL12-CXCR4 signaling (Figure 1).

## Treatment strategies for DNP

### Ammoxetine

The novel and potent serotonin and noradrenaline reuptake inhibitor (SNRI) ammoxetine, derived from duloxetine, exhibits important analgesic effects in animal models of neuropathic pain. Ammoxetine has a shorter onset time than duloxetine (Zhang et al., 2016), a lower toxicity than duloxetine, and has some hepato-protective effects (Xue et al., 2013). In the DNP rat model, the use of ammoxetine did not affect blood glucose concentration. However, ammoxetine treatment inhibited the p38MAPK and JNK signaling pathways, significantly reduced the upregulation of the microglial marker IBA-1 (thereby inhibiting microglial reactivity) and decreased IL-1β and TNF-α secretion to ameliorate DNP (Zhang T. T. et al., 2018). A phase Ⅰ clinical trial demonstrated the outstanding pharmacokinetic profile and safety of oral ammoxetine (Shen et al., 2021). In the DNP rat model, higher the dose of ammoxetine within a safe dosage range, more significant was the observed reduction in mechanical allodynia (Zhang T. T. et al., 2018). While duloxetine is used as a first-line agent for the treatment of DNP (Jiang et al., 2022), ammoxetine has been shown in several basic trials to be superior to duloxetine for treating DNP (Xue et al., 2013; Zhang et al., 2016). Common side effects of ammoxetine in clinical trials include nausea, palpitations, dry mouth, dizziness, insomnia, prolonged QT interval, pyuria, flushing, tachycardia, and hyperhidrosis (Shen et al., 2021). However, clinical trials of ammoxetine for the treatment of DNP have not yet been initiated, and further investigation is warranted.

### Photobiomodulation therapy

Photobiomodulation therapy (PBMT) can modulate cellular mitochondrial redox signaling (Hamblin, 2018) and can be applied to treat pain. It does not improve metabolic problems such as hyperglycemia and weight loss in diabetic rats. However, photobiomodulation therapy (904 nm) treatment reduced p38 phosphorylation, phosphorylation of extracellular signal-regulated kinase (ERK)1/2 proteins, p-JNK expression, and hyperalgesia (Vieira et al., 2022). PBMT also resulted in decreased expression of the microglial marker IBA-1, inhibited microglial reactivity, enhanced recovery of microglial morphology and function from insulin treatment, and prevented allodynia and hyperalgesia (Liu S. et al., 2023; Daigo et al., 2023; Marques et al., 2023; Zhang et al., 2023). A clinical trial NCT05032612 (https://clinicaltrials.gov/) applied photobiomodulation therapy to the treatment of post-endodontic pain, and the results showed that patients experienced significant pain relief, with no serious adverse effects detected. Therefore, further studies on the mechanism by which PBMT treats DNP *via* effects on microglial reactivity are needed.

### EGCG

(−)-Epigallocatechin-3-gallate (EGCG) is enriched in green tea, a popular beverage worldwide. EGCG has potent antioxidant (Higdon and Frei, 2003), anti-inflammatory (Li et al., 2020), and antitumor (Kuriyama et al., 2006) effects. EGCG can inhibit lipopolysaccharide (LPS)-induced neurotoxicity and plays a neuro-protective role (Liu et al., 2016). EGCG can also be used as a neuro-protective agent in treating neurodegenerative diseases (Pervin et al., 2018). Interestingly, EGCG supplementation can effectively reduce body weight in obese mice, alleviating diabetes (Wolfram et al., 2006; Lee M. S. et al., 2009) and reduce neural tube defects induced by gestational diabetes (Zhong et al., 2016). Both intrathecal (2 μg d^-1^) and intraperitoneal (20 mg kg^-1^ d^-1^) administration of EGCG can alleviate neuropathic pain and reduce neuro-inflammation in diabetic mice, inhibiting the reactivity of microglia and promoting IGF-1 expression in microglia (Chen et al., 2022). Thus, in future clinical studies, EGCG may be used as a novel drug to verify its effects in human DNP. Clinical studies have shown no serious adverse effects in humans taking 800 mg or less of EGCG daily, but diarrhea and headache may occur (Siblini et al., 2023). The U.S. Pharmacopoeia states that long-term high-dose supplementation with green tea extract may risk potential liver injury (Oketch-Rabah et al., 2020).

### JMT

Jinmaitong (JMT) is a traditional Chinese medicinal prescription consisting of 12 natural medicines that has been historically used to prevent and treat DNP. JMT treatment modulates microglial reactivity through inhibition of the JAK2/STAT3 signaling pathway, resulting in decreased expression of microglia markers IBA-1, CD11B, and CD68, and suppression of the NLRP3 inflammasome. JMT treatment inhibited neuro-inflammation and attenuated DNP symptoms (e.g., mechanical allodynia and hyperalgesia) in diabetic rats (Sun et al., 2019; Sun Q. et al., 2021; Wang et al., 2024). JMT targets specific signaling pathways and modulates the microglial reactivity to effectively alleviate DNP, providing a basis for investigating this preparation and its constituent actives.

### Koumine

Koumine is a compound extracted from *Gelsemium* that has anti-inflammatory and anti-neuropathic effects (Wang et al., 2019; Ye L. X. et al., 2021). Koumine treatment ameliorates DNP in rats and significantly reduces the expression of CD86, CD68, TNF-α, and IL-1β and inhibits microglial reactivity through the Notch-RBP-Jκ signaling pathway (Jin et al., 2021). Koumine is a potential drug for the treatment of DNP, and further studies are needed for its specific application in humans.

### Coenzyme Q10

The fat-soluble antioxidant coenzyme Q10 is present in most plant and animal cells and is involved in electron transfer and aerobic respiration in mitochondria. It is primarily used as a food supplement (Arenas-Jal et al., 2020), and its observed safety level in humans is 1,200 mg/day with no severe adverse effects (Hidaka et al., 2008). Coenzyme Q10 may be used as a supplement in treating diabetes, but the mechanism is currently undefined (Arenas-Jal et al., 2020). In a T2DM mouse model, coenzyme Q10 has analgesic effects in mechanical allodynia and thermal hyperalgesia. Coenzyme Q10 may relieve DNP by inhibiting the signaling pathways activated by MAPK, NF-κB, and TLR4 in the DRG and spinal cord. TLR4 is a key receptor that initiates microglial reactivity (Tanga et al., 2005); thus, its downregulation may inhibit microglial reactivity. This study also suggests that long-term use of coenzyme Q10 may play a role in preventing DNP and has better therapeutic effects when used in combination with other analgesic therapies (Zhang et al., 2013). The results of a clinical trial NCT0286546 (https://clinicaltrials.gov/) showed that coenzyme Q10 supplementation relieved chronic pain in participants to some extent without any serious adverse effect. Coenzyme Q10 may play an effective, preventive and adjunctive therapeutic role in DNP by inhibiting microglial reactivity; however, detailed mechanisms need to be further investigated.

### Electroacupuncture

Electroacupuncture (EA) improves sleep and anxiety and has a favorable antidepressant effect (Zhao et al., 2023). EA relieves neuropathic pain (Moon et al., 2020), but further trials are needed to confirm its effectiveness and safety. Receiving continuous electroacupuncture for 30 min once a day for a week has an analgesic effect in DNP (Ma et al., 2023) and can downregulate the expression of P2X7 and P2X4 in the DRG (Hu et al., 2023). EA reduced DNP-induced expression of IBA-1, BDNF, IL-1β, and TNF-α and altered microglial morphology (Qu et al., 2023). Thus, EA can alleviate DNP by regulating microglia. EA may have side effects like dizziness, gastrointestinal discomfort, and high fever, yet considered safe (He et al., 2022).

### Dexmedetomidine

Dexmedetomidine (Dex) is an α2-adrenergic receptor agonist. Dex has sedative, analgesic, anti-inflammatory, and antioxidant effects and reduces sympathetic tone and inflammatory response (Mantz et al., 2011; Li et al., 2018). Although Dex treatment did not significantly improve metabolic problems, such as body weight changes and blood glucose dysregulation in diabetic rats (Zhang et al., 2020), it relieved neuropathic pain by inhibiting P2X4 expression in the spinal cord (Kang et al., 2019). Dex (50 μg·kg^-1^) significantly reduced the mechanical withdrawal threshold (MWT) and motor nerve conduction velocity, as well as inhibited microglial reactivity and partially restored neuronal phenotype (Lu et al., 2017; Kang et al., 2019; Zhang et al., 2020). Dex also reduces DNP through the Wnt10a/β-catenin (Zhong et al., 2018) and ERK (Chen et al., 2017) signaling pathways. Common adverse reactions of Dex use are hypotension/hypertension and bradycardia (Keating, 2015).

### Quercetin

Quercetin is a natural flavonoid found in vegetables and fruits (Ay et al., 2021). Quercetin has anti-inflammatory effects and can relieve neuropathic pain through the AMP-activated protein kinase (AMPK)/MAPK pathway (Ye G. et al., 2021). Quercetin treatment blocked P2X4R expression in the DRG by acting on the P2X4R, impairing its normal function, and thereby decreasing P2X4R-mediated activation of p38MAPK in diabetic rats. Quercetin treatment (50 mg·kg^-1^day^-1^) alleviates DNP by acting on P2X4R to decrease thermal and mechanical hyperalgesia in diabetic rats (Yang et al., 2019; Wang et al., 2020). Quercetin can impede microglial reactivity and exert neuro-protective effects by inhibiting NLRP3 inflammasome activation (Han et al., 2021). Quercetin has shown marked potential as a future drug for the treatment of DNP, and further investigation of the mechanism by which quercetin modulates microglial reactivity must be further investigated.

### GLP-1RA

GLP-1 agonists (GLP-1RAs), including exenatide, liraglutide, lixisenatide, and beheneruptide, are a well-established class of drugs for the treatment of T2DM (Drucker, 2018). GLP-1RA administration can ameliorate CNS disorders by reducing microglial reactivity (Lee et al., 2018). The intracerebroventricular administration of GLP-1RA inhibits reactivity of microglia in the brain of DNP rats and relieves thermal and mechanical hyperalgesia by inhibiting activation of NLRP3 inflammatory vesicles in brain microglia (Zhang et al., 2022), and no serious adverse effects of GLP-1 agonists have been detected. Thus, treating DNP with GLP-1RA represents a potential novel research avenue.

### Taxifolin

Taxifolin, also known as dihydroquercetin, is a flavonoid present in French maritime pine, larch, milk thistle, and onions (Thuan et al., 2022). Taxifolin has antioxidant and anti-inflammatory properties and provides neuro-protection against neurodegenerative diseases (Sunil and Xu, 2019; Yang et al., 2023b). Taxifolin relieves hyperglycemia-induced neuropathic pain (Alay et al., 2022). In addition, taxifolin can inhibit TXNIP-NLRP3 axis activation by decreasing microglial ROS levels, thereby suppressing microglia-induced inflammatory responses (Iwasa et al., 2023). Taxifolin may thus achieve relief from DNP by inhibiting the microglial response to hyperglycemia. Taxifolin has a few or almost no side effects in normal cells (Das et al., 2021).

### Go-sha-jinki-gan

Go-sha-jinki-gan (GJG), a conventional herbal medicine used in humans at a standard daily dose of 7.5 g, reduces elevated blood insulin levels (Hirotani et al., 2011) and relieves chemotherapy-induced peripheral neuropathic pain (Cascella and Muzio, 2017). GJG can effectively relieve DNP (Jiang et al., 2021). GJG inhibits microglial reactivity in an experimental autoimmune encephalomyelitis (EAE) mouse model, reduces TNF-α levels, and inhibits phosphorylation of p38 in the spinal cord of EAE mice, thereby reducing CNS inflammation (Jiang et al., 2021). No adverse effects of GJG have been detected (Kishida et al., 2015). In the future, GJG could serve as a potential agent for treating DNP by inhibiting microglial reactivity.

### Metformin

Metformin is a first-line prescription drug for T2DM and effectively controls the blood glucose levels with an impressive safety profile. Its primary mechanism of action is the inhibition of hepatic gluconeogenesis (LaMoia and Shulman, 2021). Metformin reverses and blocks neuropathic pain completely in male mice and induces AMPK signaling, which is a negative regulator of MAPK signaling pathway targets (Inyang et al., 2019). Metformin treatment reduces the number of IBA-1 staining-positive microglia in the dorsal horn and inhibits microglial reactivity (Inyang et al., 2019). Thus, metformin may alleviate DNP in male mice by inhibiting the MAPK signaling pathway and suppressing microglial reactivity. The main adverse effects of metformin are gastrointestinal disturbances (Hostalek et al., 2015). The therapeutic effect of metformin is long-lasting in animal experiments (Inyang et al., 2019), whether metformin is safe and effective in relieving pain in patients with DNP remains unclear. Similarly, gender differences in the pain relief produced by metformin must be investigated in human trials.

### Osthole

Osthole is a coumarin derivative found in plants, such as *Angelica pubescens* and *Cnidium monnier*. Osthole has antitumor, antioxidant, anti-inflammatory, and neuro-protective properties (Sun M. et al., 2021; Yang et al., 2022) and can be used in the treatment of diabetes (Liang et al., 2009; Yao et al., 2018). Osthole (20 mg kg^-1^ day^-1^) treatment restored the MWT to normal levels, inhibited soluble guanylate cyclase activation and P2X4R expression in the DRG of diabetic rats, and reduced the upregulation of TNF-α, IL-1β, BDNF, and phospho-p38MAPK, thus alleviating DNP (Yuan et al., 2018). Osthole treatment reduces the levels of IL-6, IL-1β, and TNF-α, blocks NF-κB activation, reduces the expression of microglia, and inhibits microglial reactivity (Kong et al., 2019; Liu C. H. et al., 2023). Osthole has very few adverse effects (An et al., 2016). Inhibition of microglial reactivity may be the mechanism by which Osthole treats DNP, which needs to be explored in further studies.

### Dihydromyricetin

Dihydromyricetin (DHM) is a flavonoid compound that is found in abundance in *Ampelopsis grossedentata* (Hand.-Mazz.) W.T. Wang (Vitaceae) (Zhang J. et al., 2018). DHM lowers blood glucose, improves the sensitivity of the liver to insulin, and is used to treat diabetes (Le et al., 2016; Tong et al., 2020). Treatment with DHM (30 mg kg^-1^) for 14 consecutive days significantly reduced the thermal withdrawal latency (TWL) and MWT of rats with DNP, and the use of DHM reduced P2X7R expression in rats suffering from DNP and alleviated neuropathic pain and depression (Guan et al., 2019). DHM has also been shown to ameliorate microglial over-activation (Al Omran et al., 2022). No adverse effects have been reported in the clinical application of DHM (Chen et al., 2015). Thus, DHM may represent a novel drug suitable for clinical trials for DNP treatment.

### Palmatine

Palmatine, a proto-berberine alkaloid with neuro-protective, anti-inflammatory, and antioxidant effects (Long et al., 2019), is also used to treat diabetes. Treatment with palmatine (30 mg kg^-1^) for 14 consecutive days reduces MWT and TWL to alleviate DNP by reducing P2X7R expression, potentially inhibiting IL-1β and TNF-α secretion, and reducing ERK1/2 phosphorylation in the hippocampus of rats (Shen et al., 2018). Palmatine reduces TNF-, inducible nitric oxide synthase, and NF-κB immunoreactivities and inhibits microglial reactivity (Liu C. H. et al., 2023). Palmatine treatment may have the side effect of arrythmia (Tarabasz and Kukula-Koch, 2020).

### Rhodioloside

Rhodioloside is the rhizome extract of *Rhodiola rosea*, which has anti-inflammatory, antioxidant, antiplatelet, and immunomodulatory properties. Rhodioloside treatment can dramatically reduce fasting blood glucose and improve insulin resistance in diabetic rats, as well as attenuate inflammatory responses, reduce hyperalgesia, allodynia, and the upregulation of P2X7R expression (Ni et al., 2017; Zheng et al., 2021). Rhodioloside modulates the microglial reactivity and is used to alleviate neuroinflammation (Wang et al., 2018). No adverse effects have been detected during its clinical application (Zhang et al., 2012). Therefore, rhodioloside has a promising future in DNP treatment *via* inhibition of microglial reactivity.

### Nanoparticle-encapsulated curcumin

Curcumin is a diketone compound extracted from the rhizome of turmeric with anti-inflammatory, antioxidant, anticancer, and hypolipidemic effects (Park et al., 2021). Nanoparticle-encapsulated curcumin has increased targeted delivery ability, bioavailability, and stability compared to un-encapsulated curcumin (Guo et al., 2016). Nanoparticle-encapsulated curcumin reduced the expression level of P2Y_12_R and slowed down mechanical and thermal hyperalgesia in diabetic rats (Jia et al., 2017). Curcumin inhibited microglial pyroptosis and pro-inflammatory responses (Ran et al., 2021). Nano curcumin treatment reduces pain levels and lowers fasting blood glucose in patients with DNP and is well tolerated (Asadi et al., 2019). More research is needed in the future to investigate the mechanisms by which curcumin acts on microglia.

### Cilnidipine

Cilnidipine is a Ca^2+^ channel blocker that inhibits L/N-type Ca^2+^ channels (Shete, 2016). A study has shown that cilnidipine improves insulin sensitivity (Yagi et al., 2003). In addition, cilnidipine inhibited endogenous P2X7R expression in microglia and suppressed IL-1β release from microglia while relieving neuropathic pain (Yamashita et al., 2021). Thus, cilnidipine may alleviate DNP by inhibiting microglial reactivity while improving diabetic symptoms. Cilnidipine may provide a new research direction for DNP treatment.

### Resveratrol

Resveratrol is a plant antitoxin found mainly in vegetables, fruits, grains, and wine with antioxidant, anti-inflammatory, and antitumor properties (Huang et al., 2020). Resveratrol can treat diabetes by improving insulin resistance (Wong and Howe, 2018), enhancing glucose uptake and metabolism (Sadi et al., 2015), and restoring the secretory function of insulin cells (Lee J. H. et al., 2009). Resveratrol at a dose of 200 mg daily for 24 weeks can control blood glucose level and reduce inflammation and oxidative stress associated with diabetes (Mahjabeen et al., 2022). Resveratrol has considerable neuro-protective ability (Rahman et al., 2020), and can inhibit inflammatory damage by inhibiting microglial reactivity directly and secondary glial reactivity (Mota et al., 2020). Resveratrol has been proven to relieve DNP in mouse model (Sharma et al., 2007). Thus, resveratrol may treat DNP by inhibiting microglial reactivity; however, the exact mechanism requires further research.

### Botulinum toxin type A

Botulinum toxin is an exotoxin originating from the bacterium *Clostridium botulinum*. Botulinum toxin type A is used in medical cosmetology and in the treatment of autonomic nervous disorders and movement disorders, and has a favorable effect when included in the treatment of chronic pain (Matak et al., 2019; Yoshida, 2021). Botulinum toxin type A injection therapy relieves DNP with few adverse effects (Salehi et al., 2019; Wang et al., 2021). Injection of botulinum toxin type A (0.18U) inhibits microglial reactivity and prominently downregulates the mRNA expression of TLRs, especially TLR5 and TLR2, which is closely associated with reducing mechanical pain hypersensitivity behaviors in mice (Chen et al., 2021). Thus, botulinum toxin type A provides a novel therapeutic approach for DNP, with a dose of 300–600 U applied for medical indications (Berry and Stanek, 2012).

### N-palmitoyl-D-glucosamine

N-palmitoyl-D-glucosamine (PGA) is a natural molecule produced by bacteria such as *Rizhobium leguminosarum* (Philip-Hollingsworth et al., 1997) with analgesic and anti-inflammatory effects (Cordaro et al., 2019). PGA acts as a TLR4 antagonist and antagonizes LPS at TLR4 to inhibit LPS-triggered NF-κB activation. PGA can prevent LPS-induced pro-inflammatory cytokine release and attenuate neuropathic pain (Iannotta et al., 2021). TLR4 is a key receptor that initiates microglial reactivity; thus, downregulation of TLR4 inhibits microglial reactivity. Oral PGA inhibits microglial reactivity and provides pain relief (Gugliandolo et al., 2023). The adverse effects of using PGA are not well understood. PGA may play a therapeutic role in DNP by inhibiting microglial reactivity, but the exact mechanism requires further investigation (Table 1).

**TABLE 1:** Latest and potential medications as well as treatments acting on microglia for DNP.

Name	Mechanism	Treatment results	Adverse reaction
Ammoxetine	Inhibition of p38MAPK, JNK signaling pathway and microglia reactivity	Improves DNP	Nausea, dizziness, palpitations, tachycardiaetc.
Photobiomodulation therapy	Reduces P38 phosphorylation and p-JNK expression, inhibits microglia reactivity	Prevents allodynia and hyperalgesia	---
EGCG	Inhibition of the reactivity of microglia and maintenance of microglia IGF-1 expression	Relieves diabetes and reduces DNP	Diarrhea, headaches
Jinmaitong	Inhibition of the JAK2/STAT3 signaling pathway	Improves DNP	---
Koumine	Inhibits microglia response	Improves DNP	---
Coenzyme Q10	Inhibits the signaling pathways activated by MAPK, NF-κB, and TLR4 in DRG and spinal cord	Analgesic effects on mechanical allodynia and thermal hyperalgesia	---
Electroacupuncture	Downregulation of P2X7 and P2X4 expression in DRG and inhibition of microglia reactivity	It has antidepressant properties and reduces DNP	Dizziness, gastrointestinal distress and high fever
Dexmedetomidine	Inhibition of P2X4 expression and microglia reactivity	Multiple pathways to relieve DNP	Hypotension, hypertension and bradycardia
Quercetin	Inhibition of P2X4 expression and microglia reactivity	Multiple pathways can be used to relieve neuropathic pain	---
GLP-1RA	Inhibition of NLRP3 inflammatory vesicle activation in brain microglia	Improves DNP	---
Taxifolin	Reduces the activation of TXNIP-NLRP3 axis, thereby inhibiting microglia response to hyperglycemia	Relieves hyperglycemia-induced neuropathic pain	---
Go-sha-jinki-gan	Inhibition of microglia reactivity and phosphorylation of P38	Reduces high insulin levels in the blood and improves DNP	---
Metformin	Inhibits microglia reactivity and induces AMPK signaling	It has a therapeutic effect on diabetes and reduces DNP	Gastrointestinal disturbances
Osthole	Inhibition of P2X4R expression and microglia reactivity	Adjuvant treatment of diabetes and reduction of DNP	---
Dihydromyricetin	Reduces P2X7R expression and ameliorates microglia overactivation	It may lower blood glucose and improve DNP	---
Palmatine	Inhibition of P2X7R expression and microglia reactivity	Be used to treat diabetes and improve DNP	Arrhythmia
Rhodioloside	Inhibition of P2X7R expression and microglia response	Lowers blood glucose and improves insulin sensitivity, reduces DNP	---
Nanoparticle-Encapsulated Curcumin	Reduces expression of P2Y_12_R and inhibits microglia pyroptosis	Improves DNP	---
Cilnidipine	Inhibits endogenous expression of P2X7R in microglia and suppresses IL-1β release from microglia	relieves neuropathic pain	---
Resveratrol	Inhibition of microglia reactivity and neuroprotection-mediated secondary glial reactivity	Improves insulin resistance and DNP	---
Botulinum toxin type A	Significantly downregulates mRNA expression of TLRs and inhibits microglia reactivity	Improves DNP	---
N-palmitoyl-D-glucosamine	Acts on TLR4 to antagonize LPS and inhibits microglia reactivity	Improves pain	---

## Summary and conclusion

DNP is among the most serious complications affecting patients with diabetes. Current DNP treatment options are associated with high costs to patients and inadequate results, creating a considerable burden for patients and health services alike. During DNP development, microglia are reactive, and inhibition of P2X4R, P2X7R, P2Y_12_R, TLRs, and other microglial surface receptors involved in the intracellular signaling pathway associated with microglial reactivity, can provide a good analgesic effect. Inflammatory factors, biological factors, and chemokines are also involved in the development of DNP, and studies have suggested that specific blockade of these biologically active factors can significantly alleviate pain. Although the signaling pathways of microglia involved in DNP development are not fully understood, drugs and treatments are available that have been subjected to basic experiments to validate their DNP-relieving effects. Drugs such as ammoxetine have demonstrated superiority over the current first-line therapeutic drugs, but majority of the targeted drugs and therapeutic methods have not entered clinical trials to validate their therapeutic efficacy. In the future, advancement of these targeted drugs and treatments to the clinical trial stage is required to offer better treatment outcomes for patients with DNP.

## Figures and Tables

**Table:** 

**AMPK**	AMP-activated protein kinase
**ATPase**	adenosine triphosphatase
**BDNF**	Brain-Derived Neurotrophic Factor
**CGRP**	calcitonin gene-related peptide
**CNS**	central nervous system
**CSF-1**	Colony-stimulating factor 1
**DNP**	diabetic neuropathic pain
**DRG**	dorsal root ganglion
**ERK**	extracellular signal-regulated kinase
**IBA-1**	ionized calcium binding adapter molecule 1
**IGF-1**	Insulin-Like Growth Factor I
**IL-1**	interleukin-1
**IL-1β**	interleukin-1β
**IL-6**	interleukin-6
**IL-18**	interleukin-18
**IL-18R**	interleukin-18
**iNOS**	inducible nitric oxide synthase
**JAK/STAT**	Janus kinase/signal transducer, and the activator of transcription
**JNK**	c-Jun N-terminal kinase
**LPS**	Lipopolysaccharide
**MAPK**	Mitogen-activated protein kinase
**NF-κB**	nuclear factor κB
**NLRP3**	NLR family pyrin domain containing 3
**nSTZ**	neonatal administration of STZ
**P2XR**	P2X receptor
**P2X7R**	P2X7 receptor
**P2X4R**	P2X4 receptor
**PKC**	Protein Kinase C
**ROS**	reactive oxygen species
**SNRI**	Serotonin and Noradrenaline Reuptake Inhibitors
**STZ**	streptozotocin
**TLRs**	toll-like receptors
**TNF-α**	tumor necrosis factor-α
